# Electrostatics and hydrophobicity in the dynamics of intrinsically disordered proteins

**DOI:** 10.1140/epje/s10189-023-00383-7

**Published:** 2023-12-21

**Authors:** Renee Vancraenenbroeck, Hagen Hofmann

**Affiliations:** 1 Department of Chemical and Structural Biology, Weizmann Institute of Science, Herzl St. 234, 76100 Rehovot, Israel; 2 Present Address: Present Address: Department of Structural and Molecular Biology, University College London, Darwin Building, 107 Gower Street, London, WC1E 6BT UK

## Abstract

Internal friction is a major contribution to the dynamics of intrinsically disordered proteins (IDPs). Yet, the molecular origin of internal friction has so far been elusive. Here, we investigate whether attractive electrostatic interactions in IDPs modulate internal friction differently than the hydrophobic effect. To this end, we used nanosecond fluorescence correlation spectroscopy (nsFCS) and single-molecule Förster resonance energy transfer (FRET) to quantify the conformation and dynamics of the disordered DNA-binding domains Myc, Max and Mad at different salt concentrations. We find that internal friction effects are stronger when the chain is compacted by electrostatic attractions compared to the hydrophobic effect. Although the effect is moderate, the results show that the heteropolymeric nature of IDPs is reflected in their dynamics.

**Graphical abstract:**

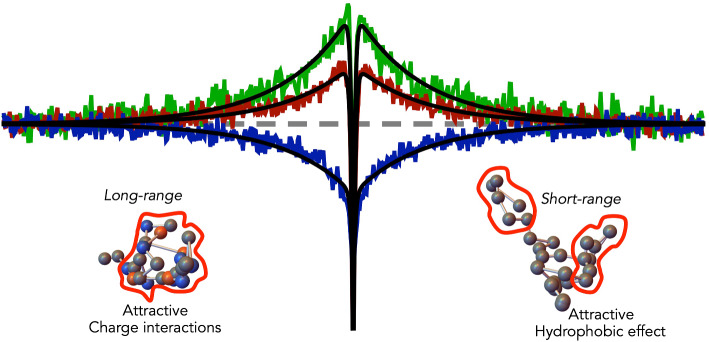

## Introduction

The dimensions of intrinsically disordered proteins (IDPs) depend on a balance between different types of interactions. Among them, electrostatic interactions [1–3] and the hydrophobic effect [4, 5] are particularly relevant due to their rather nonspecific nature, lacking geometric constraints. Understanding how these interaction types affect IDPs has been critical for predicting their response to changes in solvent conditions such as salt concentrations or denaturants. For instance, the bare number of acidic and basic amino acids dictates whether an IDP behaves as a polyelectrolyte or as a polyampholyte [1, 2, 4, 6–8], thus allowing predictions of the salt-dependence of their dimensions. Similarly, the patterning of charges along the chain can modulate chain dimensions even at identical overall charge compositions [9]. Quantifications of this effect via the charge patterning parameter *κ* [9] or the sequence charge decoration metric (SCD) [10, 11] nowadays belong to the standard repertoire in analyzing IDPs. However, much less is known about how these factors affect the dynamics of IDPs. In proteins, deviations from long-standing models such as Kramers’ reaction rate theory [12] and the Rouse model of polymer dynamics [13] are consistently found, ranging from the diffusive dynamics of IDPs and unfolded proteins [14–16], to protein folding reactions [17–20] and ligand dissociation [21], to transition path times of folding reactions [22]. Potential sources for some of these deviations include elementary bond rotations [23–26], interactions between buried residues [21, 27–29], and electrostatic effects [22, 30]. However, particularly for the comparatively simple dynamics of isolated IDPs, modifications of the Rouse model by an additional fitting parameter to account for ‘internal friction’ effects [23, 26, 27, 31, 32] have been sufficient to describe experimental data [33]. In these modifications, four parameters describe the dynamics of an IDP: chain length, monomer size, chain dimension, and internal friction.

Here, we go one step beyond this picture. We ask whether the type of interactions that dictate chain dimensions affect the reconfiguration time of IDPs differently. Specifically, we determined the internal friction timescales of three homologous IDPs, the DNA-binding domains of the transcription factors Myc, Max, and Mad, at different salt and denaturant concentrations. We indeed found that internal friction depends on the types of interactions that dominate chain dimensions. The result is a first step toward unraveling the various energetic contributions to internal friction in disordered proteins. We therefore hope that this work will help establishing an analytical toolset of IDP dynamics that will be comparable in power to current predictions of thermodynamic properties.

## Methods

### Confocal microscope

Single-molecule fluorescence measurements were performed with a MicroTime 200 confocal microscope (PicoQuant) equipped with an Olympus IX73 inverted microscope. We used linearly polarized light from a 485 nm diode laser (LDH-D-C-485, PicoQuant) at a repetition rate of 40 MHz to excite individual labeled proteins, while they were diffusing through the confocal volume in our microscope. Nanosecond FCS experiments were performed with a continuous excitation. The excitation beam was guided via a major dichroic mirror (ZT 470–491/594 rpc, Chroma) through the microscope objective (60 × 1.2NA Olympus) into the sample. The sample was placed in a home-made cuvette (50 µl sample volume) made from a 25 mm diameter round quartz cover slip (ESCO Optics) and a borosilicate glass cloning cylinder (6 mm diameter, Hilgenberg). All measurements were performed by exciting the donor dye with a laser power of 100 μW, measured at the back aperture of the objective. Photons emitted from the sample were collected by the same objective. After passing the major dichroic mirror (ZT 470-491/594 rpc, Chroma), the residual excitation light was removed by a long-pass filter (BLP01-488R, Semrock). The remaining emission light was then focused on a pinhole with diameter of 100 µm. For the determination of smFRET histograms, the fluorescence was then separated into donor and acceptor photons using a dichroic mirror (T585 LPXR, Chroma) and focused onto two single-photon avalanche diodes (SPAD, Perkin Elmer) after passing through additional bandpass filters for donor (FF03-525/50, Semrock) and acceptor (FF02-650/100, Semrock) photons. For nsFCS experiments, the emission light was first split according to the polarization of the photons using a polarization beam splitter. Afterward, each polarization was separated into donor and acceptor photons and focused onto separate SPADs using the same filters as described above. For smFRET measurements, the arrival time of every detected photon was recorded with a HydraHarp 400 M time-correlated single-photon counting (TCSPC) module (PicoQuant).

### Single-molecule FRET experiments

The proteins Myc, Max, Mad, or modified Myc (ΔMyc) were expressed, purified and labeled as described in Vancraenenbroeck et al. [4]. The sequences and labeling positions of the proteins can be found in Table 1. For smFRET experiments, the samples were diluted to a concentration of approximately 50–100 pM in (20 mM Tris–HCl pH 8) buffer at the appropriate concentration of KCl, urea, or GdmCl (Pierce). Our buffer alone causes an ionic strength of 0.01 M, which has been taken into account for all fits with polyampholyte theory and the Rouse model. To prevent surface adhesion of the proteins and to maximize photon emission, 0.001% Tween 20 (Pierce) and 100 mM β-mercaptoethanol were included in the buffer and cuvettes were coated with poly-l-lysine at low ionic strengths (below 0.5 M KCl or 0.3 M GdmCl). All measurements were performed at 23 °C. The acquisition time for single-molecule FRET measurements was 10 min to 15 min, and the acquisition time for nsFCS measurements was 12 to 26 times 1 h.

**Table 1:** Aligned amino acid sequences

Protein	Sequence	
MYC	GPG**C**SGNVKRRTHNVLERQRRNELKRSFFALRDQIPELEN	NEKAPKVVILKKATAYILSVQAEEQKLISEEDLLRKRREQLKHKLEQLGS**C**
	ϕ + + + ϕϕ− + + + −ϕ + + ϕϕ ϕ +− ϕ –ϕ	− + + ϕϕϕϕ + + ϕϕ ϕ – + ϕϕ –−ϕϕ + + + + − ϕ + + ϕ− ϕ
MAX	GPG**C**SGADKRAHHNALERKRRDHIKDSFHSLRDSVPSLQG	EKASRAQILDKATEYIQYMRRKNHTHQQDIDDLKRQNALLEQQVRALGS**C**
	− + + ϕ− + + + +− ϕ +− ϕ ϕ +− ϕ ϕ	−+ + ϕϕ− +—ϕ ϕ + + + −ϕ–ϕ + + ϕϕ− ϕ + ϕ
MAD	GPG**C**SGSSSRSTHNEMEKNRRAHLRLSLEKLKGLVPLGPE	SSRHTTLSLLTKAKLHIKKLEDSDRKAVHQIDQLQREQRHLKRQLEKLGS**C**
	+ -ϕ- + + + ϕ + ϕ ϕ- + ϕ + ϕϕ ϕ−	+ ϕ ϕϕ + + ϕ ϕ + + ϕ–—+ + ϕ ϕ- ϕ +—+ ϕ + + ϕ- + ϕ
ΔMYC	GPG**C**SGNSKRRTHNGSERQRRNEGKRSSGASRDQGPESEN	NEKAPKGSGSKKATAYGSSGQAEEQKSGSEEDSGRKRREQSKHKGEQSGS**C**
	+++ −+ ++ − ++ +− − −	−+ + ++ −− + −−− ++++− + + −

The charges at pH 8 are indicated below the respective amino acids with + (R, K) and—(D, E), bulky hydrophobic amino acids (V, I, L, M, F) are indicated with ϕ. Cysteine to serine mutations in MAD are double underlined. Mutated residues in ΔMYC are underlined and all labeling positions are in bold

### Burst identification and data analysis

Fluorescence bursts from individual molecules were determined by combining successive photons separated by inter photon times of < 100 μs. Identified bursts were corrected for background, differences in quantum yield of donor and acceptor, different collection efficiencies in the detection channels, cross-talk, and direct acceptor excitation as described previously [34, 35]. Bursts were retained if the total number of photons detected was $T>50$ (low GdmCl and KCl concentrations) or $T>100$ (high GdmCl and KCl concentrations). The corrected photon numbers of donor (${n}_{D}$) and acceptor (${n}_{A}$) after donor exciation were used to compute the FRET efficiency of the burst via

1 $$ E = \frac{{n_{A} }}{{n_{A} + n_{D} }}. $$

Since the identified bursts also contain molecules for which the acceptor bleached during the transit trough the confocal spot, thus masking the true transfer efficiency, we further cleaned the FRET histograms by these events [36]. For a burst with ${n{^\prime}}_{D}$ donor photons with the arrival times ${t}_{D,1}\dots {t}_{D,{n{^\prime}}_{D}}$ and ${n{^\prime}}_{A}$ acceptor photons with the arrival times ${t}_{A,1}\dots {t}_{A,{n{^\prime}}_{A}}$, the average arrival times are given by $\langle {t}_{D}\rangle ={n{^\prime}}_{D}^{-1}{\sum }_{i}{t}_{D,i}$ and $\langle {t}_{A}\rangle ={n}_{A}{{^\prime}-1}{\sum }_{i}{t}_{A,i}$. Here, the prime indicates the uncorrected photon counts. The burst asymmetry is defined by ${\alpha }_{DA}=\langle {t}_{D}\rangle -\langle {t}_{A}\rangle$. If the acceptor dye bleaches, we find ${\alpha }_{DA}>0$. Taking shot noise into account, the distribution of ${\alpha }_{DA}$ has a standard deviation given by

2 $$ \sigma_{DA} = \frac{T}{2\sqrt 3 }\left( {\frac{1}{{n{^\prime}_{D} }} + \frac{1}{{n{^\prime}_{A} }}} \right)^{1/2} . $$

To eliminate molecules with a bleached acceptor, we excluded all molecules for which ${\left|{\alpha }_{DA}\right|>\sigma }_{DA}$. Finally, the mean FRET efficiency was determined by fitting the FRET efficiency histogram with a combination of a log-normal distribution and a Gaussian peak or two Gaussian peaks. The log-normal distribution describes bursts with FRET efficiencies close to zero, which results from molecules that lack an active acceptor dye. The second peak describes the FRET efficiency distribution of doubly labeled molecules.

### Determination of donor–acceptor distances from mean FRET efficiencies

We determined the average donor acceptor distance ${R}_{DA}$ using

3 $$ E = \mathop \int \limits_{{r_{c} }}^{L} E\left( r \right)P\left( r \right){\text{d}}r/\mathop \int \limits_{{r_{c} }}^{L} P\left( r \right){\text{d}}r. $$

Here, $L={N}_{\mathrm{bonds}}b$ with $b=0.38\, \mathrm{nm}$ is the distance between two successive C_α_-atoms, ${N}_{\mathrm{bonds}}=N+l$ is the effective number of bonds between donor and acceptor fluorophores that includes an estimate for the contribution of the dye linker $l=9$, ${r}_{c}=0.1\, \mathrm{nm}$, $E\left(r\right)={R}_{0}^{6}/\left({R}_{0}^{6}+{r}^{6}\right)$ is the Förster equation where ${R}_{0}=5.4\, \mathrm{nm}$ is the Förster distance of our dye pair, and $r$ is the donor–acceptor distance. We followed recent approaches [37] and used the self-avoiding polymer chain model (SAW) [38] as an estimate for $P\left(r\right)$

4 $$ P\left( r \right) = 4\pi a\left( \nu \right)x^{2 + \varepsilon } \exp \left[ { - b\left( \nu \right)x^{\delta } } \right], $$

with $\nu =\mathrm{ln}{R}_{DA}/\mathrm{ln}{N}_{\mathrm{bonds}}$ being the length scaling exponent of the chain. The coefficients $a\left(\nu \right)$ and $b\left(\nu \right)$ are the solutions of the coupled system

5 $$\begin{aligned} 1 &= 4\pi a\left( \nu \right)b\left( \nu \right)^{{ - \frac{3 + \varepsilon }{\delta }}} {\Gamma }\left( {\frac{3 + \varepsilon }{\delta }} \right)\delta^{ - 1} \;{\text{and}}\;\\ 1 &= 4\pi a\left( \nu \right)b\left( \nu \right)^{{ - \frac{5 + \varepsilon }{\delta }}} {\Gamma }\left( {\frac{5 + \varepsilon }{\delta }} \right)\delta^{ - 1}\end{aligned}$$

where $\Gamma \left(z\right)$ is Euler’s Gamma function. In addition, $x=r/{a}_{K}$ with ${a}_{K}=0.55\, \mathrm{nm}$ as average Kuhn length [37, 39] and the relations $\varepsilon =1/6\nu$ and $\delta =1/\left(1-\nu \right)$. Equation (4) was solved numerically using Mathematica 10.3 to determine the unknown donor–acceptor distance ${R}_{DA}$. To check that the resulting donor–acceptor distances are not model dependent, we alternatively determined ${R}_{DA}$ using the most simple polymer model, the Gaussian chain model, given by

6 $$ P\left( r \right) = 4\pi r^{2} \left( {\frac{3}{{2\pi R_{DA}^{2} }}} \right)^{\frac{3}{2}} \exp \left( { - \frac{{3r^{2} }}{{2R_{DA}^{2} }}} \right). $$

We show the average of both values and the deviation between the models as a colored band in all figures.

### Polyampholyte theory

We used the polyampholyte theory of Higgs and Joanny [6] that we previously extended [4] to describe IDPs at low and high ionic strengths. The force balance is given by

7 $$ 0 = \alpha^{5} - \alpha^{3} - \frac{{\omega_{2} }}{{\alpha^{3} }} - \sqrt {\frac{{6N_{{{\text{bonds}}}} }}{{\pi^{3} }}} \omega_{1} + \frac{\alpha }{{b^{2} N_{{{\text{bonds}}}} }}\left[ {l_{B}^{2} \left( {f + g} \right)^{2} \frac{{{\Lambda }_{a} }}{2} - l_{B} \left( {f - g} \right)^{2} {\Lambda }_{e} } \right] $$

With the expansion parameter $\alpha ={r}_{DA}/b\sqrt{{N}_{\mathrm{bonds}}}$ and $b=0.38\, \mathrm{nm}$. The solution of Eq. (7) is the equilibrium expansion parameter $\alpha {^\prime}={R}_{DA}/b\sqrt{{N}_{\mathrm{bonds}}}$. The fractions of positively and negatively charged residues and dyes are given by f and g. Here, ${\omega }_{1}$ and ${\omega }_{2}$ are the two-body and three-body interaction terms, respectively. The attractive (${\Lambda }_{a}$) and repulsive (${\Lambda }_{e}$) electrostatic terms are given by

8 $$ \begin{aligned} & {\Lambda }_{a} = \frac{{N_{{{\text{bonds}}}}^{2} }}{{2\kappa_{0}^{4} }}\\&\quad\left[ { - 27 + 2\kappa_{0} \left( {9\sqrt {\frac{6}{\pi }} - 6\kappa_{0} + 2\sqrt {\frac{6}{\pi }} \kappa_{0}^{2} } \right)}\right.\\&\quad\left.{ - 3e^{{2\kappa_{0}^{2} /3}} \left( {2\kappa_{0}^{2} - 9} \right){\text{erfc}}\left( {\sqrt{\frac{2}{3}}  \kappa_{0} } \right)} \right] \\ & {\Lambda }_{e} = 4\alpha b\left( {\frac{{\sqrt {N_{{{\text{bonds}}}} } }}{{\kappa_{0} }}} \right)^{5} \left[ {12\left( {6\sqrt {\frac{6}{\pi }} \kappa_{0} - \kappa_{0}^{2} - 18} \right) }\right.\\&\quad\quad\left. {+ e^{{\kappa_{0}^{2} /6}} \left( {216 - 24\kappa_{0}^{2} + \kappa_{0}^{4} } \right){\text{erfc}}\left( {\frac{{\kappa_{0} }}{\sqrt 6 }} \right)} \right] \\ \end{aligned} $$

with the unit-less inverse screening length ${\kappa }_{0}=\kappa \alpha b\sqrt{{N}_{\mathrm{bonds}}}$. As usual, the Bjerrum length is given by ${l}_{B}={q}^{2}/\left(4\pi {\epsilon }_{0}{\epsilon }_{r}kT\right)$ and the inverse Debye-screening length $\kappa =\sqrt{8\pi {l}_{B}I}$. Here, $q$ is the elementary charge, ${\epsilon }_{0}$ is the permittivity of the vacuum, ${\epsilon }_{r}=80$ is the dielectric constant of water, $I$ is the ionic strength of the solution, and $k$ is the Boltzmann constant. To allow an expansion of the chains with increasing ionic strength and denaturant concentration, we assume a linear dependence of the two-body interaction term of the form

9 $$ \omega_{1} = A_{i} I + B\;{\text{or}}\;  \omega_{1} = A_{i} I + A_{u} + B $$

in the case with urea, where ${A}_{i}$, ${A}_{u}$ and $B$ being fitting parameters and ${c}_{u}$ the urea concentration. The index $i$ indicates the type of salt and denaturant. A global fit of all data sets, including a variety of different mono-valent salts (LiCl, NaCl, KCl, and GdmCl), has been published previously [4] and here we only report the parameters relevant for this study in Table 2. Free energies of the chains at the average donor–acceptor distance ${R}_{DA}$ were obtained by integrating Eqs. (7–8). The resulting free energies are given by

$$ F_{{{\text{elastic}}}} = \alpha^{\prime 2} - 2\ln \alpha^{\prime } $$

$$ F_{{{\text{two}}\;{\text{body}}}} = 2\sqrt {\frac{{2N_{{{\text{bonds}}}} }}{{3\pi^{3} }}} \frac{{\omega_{1} }}{{\alpha^{\prime 3} }} $$

$$ F_{{{\text{three}}\;{\text{body}}}} = \frac{{\omega_{2} }}{{3\alpha^{\prime 6} }} $$

10 $$\begin{aligned} & {F}_{\mathrm{electrostatic}}=\frac{1}{12{\sqrt{\pi }{N}_{\mathrm{bonds}}b}^{6}{\alpha {^\prime}}^{3}{\kappa }^{5}}{l}_{B}\left\{-3456{\sqrt{\pi }\left(f-g\right)}^{2}\right.\\&\quad\left.+9\left[3\sqrt{\pi }{l}_{B}{\left(f+g\right)}^{2}+128\sqrt{6}b{\left(f-g\right)}^{2}{N}_{\mathrm{bonds}}^{2}{\alpha }{^\prime}\right]\kappa \right.\\&\quad\left. +18b\sqrt{{N}_{\mathrm{bonds}}}\alpha {^\prime}\right.\\&\quad\left.\left[-\sqrt{6}{\left(f+g\right)}^{2}{l}_{B}-16b\sqrt{{N}_{\mathrm{bonds}}\pi }{\left(f-g\right)}^{2}\alpha {^\prime}\right]{\kappa }^{2}\right.\\&\quad\left.+2{b}^{2}{N}_{\mathrm{bonds}}{\alpha {^\prime}}^{2}\left[9\sqrt{\pi }{\left(f+g\right)}^{2}{l}_{B}\right.\right.\\&\quad\left.\left.+16\sqrt{6}b{\left(f-g\right)}^{2}\sqrt{{N}_{\mathrm{bonds}}}\alpha {^\prime}\right]{\kappa }^{3}\right.\\&\quad\left.-8\sqrt{6}{b}^{3}{\left(f+g\right)}^{2}{l}_{B}{\alpha {^\prime}}^{3}\sqrt{{N}_{\mathrm{bonds}}^{3}}{\kappa }^{4}\right.\\&\quad\left.+9\sqrt{\pi }{e}^{\frac{1}{6}{b}^{2}{N}_{\mathrm{bonds}}{\alpha {^\prime}}^{2}{\kappa }^{2}}\left[-3{e}^{\frac{1}{6}{b}^{2}{N}_{\mathrm{bonds}}{\alpha {^\prime}}^{2}{\kappa }^{2}}\right.\right.\\&\quad\left.\left.{\left(f+g\right)}^{2}{l}_{B}\kappa \mathrm{erfc}\left(\sqrt{\frac{2{N}_{\mathrm{bonds}}}{3}}b\alpha {^\prime}\kappa \right)\right.\right.\\&\quad\left.\left.-32{\left(f-g\right)}^{2}\left({b}^{2}{N}_{\mathrm{bonds}}{\alpha {^\prime}}^{2}{\kappa }^{2}-12\right)\kappa \mathrm{erfc}\right.\right.\\&\quad\left.\left.\left(\sqrt{\frac{{N}_{\mathrm{bonds}}}{6}}b\alpha {^\prime}\kappa \right)\right]\right\}\end{aligned}$$

where $\alpha {^\prime}={R}_{DA}/b\sqrt{{N}_{\mathrm{bonds}}}$ is the equilibrium expansion parameter.

**Table 2:** Parameters obtained from global fits of the solute-driven expansion and collapse with Eqs. (7–9)

	Myc	Max	Mad	ΔMyc
${\omega }_{2}$	12.1 ± 0.10	17.2 ± 0.7	18.0 ± 1.0	12.1 ± 0.10
$B$	2.20 ± 0.05	0.67 ± 0.08	1.34 ± 0.12	2.31 ± 0.06
${A}_{\mathrm{urea}}$	1.35 ± 0.02	1.21 ± 0.02	1.46 ± 0.054	0.96 ± 0.03
${A}_{\mathrm{GdmCl}}$	2.23 ± 0.05	1.92 ± 0.04	2.22 ± 0.07	1.71 ± 0.05
${A}_{\mathrm{KCl}}$	− 1.60 ± 0.03	− 1.02 ± 0.02	− 2.10 ± 0.06	− 0.25 ± 0.05

### Nanosecond fluorescence correlation spectroscopy (nsFCS) analysis

Nanosecond FCS experiments were performed at single-molecule concentrations (50 -100 pM), which allowed us to exclusively select molecules with an active acceptor for the analysis. To reach a sufficient number of photons for high-quality nsFCS curves, each experiment required a total measurement time of 12–26 h. For each experiment, bursts were identified (see Burst identification), and the resulting FRET histograms were fitted with a combination of a log-normal (donor-only) and a Gaussian peak to determine the mean FRET efficiency $\langle E\rangle$ of the disordered ensemble. The mean FRET efficiency and the width $\upsigma$ of the FRET efficiency distribution were then used to determine the FRET efficiency interval $\Delta =\langle E\rangle \pm 2\sqrt{2\mathrm{ln}2}\sigma$. Only bursts within this interval were used to calculate the auto- and cross-correlation functions ${g}_{ij}(\tau )$. Here, the subscripts ($i,j$) refer to donor ($D$) and acceptor ($A$) molecules. The three correlation functions ${g}_{DD}(\tau )$, ${g}_{AA}(\tau )$, and ${g}_{AD/DA}(\tau )$ were globally fitted using [40]

11 $$\begin{aligned} g_{ij} \left( \tau \right) &= 1 + \frac{1}{{n_{ij} }}\left( {1 - c_{ij}^{A} e^{{ - |{t - t_{0} }| /\tau_{A} }} } \right)\\ &\quad\left( {1 \pm c_{ij}^{C} e^{{ - | {t - t_{0} } | /\tau_{C} }} } \right)\left( {1 + c_{ij}^{T} e^{{ - | {t - t_{0} } |/\tau_{T} }} } \right)\end{aligned} $$

The three terms in brackets describe photon antibunching ($A$), conformational dynamics ($C$), and triplet blinking of the dyes ($T$). In addition, ${n}_{ij}$ is the effective number of molecules in the confocal volume and ${c}_{ij}^{A}$, ${c}_{ij}^{C}$, and ${c}_{ij}^{T}$ are amplitudes. The $\pm$ sign indicates the opposite amplitudes of the decay due to distance dynamics in the autocorrelation functions ($+$) compared to the cross-correlation function ($-$). The antibunching time ${\tau }_{A}$, the correlation time ${\tau }_{C}$ and the time origin ${t}_{0}$ were global fitting parameters. To extract the proper reconfiguration times of the dynamics, we used a model in which we assume that the donor–acceptor distances diffuse in a potential of mean force $V\left(r\right)$ given by the determined donor–acceptor distribution via $V\left(r\right)=-\mathrm{ln}P\left(r\right)$. Gopich et al. showed that the correlation time is given by [41]with

12 $$ \tau_{C} = D^{ - 1} \mathop \int \limits_{{r_{c} }}^{L} P\left( r \right)^{ - 1} \left[ {\mathop \int \limits_{{r_{c} }}^{r} \delta \tilde{n}\left( \rho \right)P\left( \rho \right){\text{d}}\rho } \right]^{2} {\text{d}}r/\mathop \int \limits_{{r_{c} }}^{L} \delta \tilde{n}\left( r \right)^{2} P\left( r \right){\text{d}}r $$

is the distance-dependent fluctuation in the photon count rate. Here, $P\left(r\right)$ is the donor–acceptor distance distribution, $D$ is the intra-chain diffusion coefficient that characterizes the timescale of distance fluctuations, and ${r}_{c}=0.1\, \mathrm{nm}$ is a lower cutoff to account for the excluded volume of our dyes. Knowing $D$ and $P\left(r\right)$ then fully characterize the dynamics of an IDP. The goal is therefore to compute $D$ assuming a suitable model of the distance distribution. Since ${\tau }_{C}$ is identical for all correlation functions, it suffices to only consider the photon-rates $\widetilde{n}$ of one of the dyes, the donor in our case ($\widetilde{n}={\widetilde{n}}_{D}$). The term $\delta \widetilde{n}\left(r\right)=\widetilde{n}\left(r\right)-\langle \widetilde{n}\rangle$ can then be computed from the kinetic photo-physical scheme of excited states and ground states in a two-color FRET system. Neglecting the possibility that donor and acceptor can simultaneously populate their excited states and using the base (DA, D*A, DA*) where D and A indicate the ground state of donor and acceptor, respectively, and the asterisk indicates the excited states of the dyes; the populations of these photo-physical states expressed in the vector $$\mathbf{p}=\left(\begin{array}{ccc}{p}_{DA}& {p}_{{D}^{*}A}& {p}_{D{A}^{*}}\end{array}\right)$$ are given by a linear and homogeneous differential equation system

$$ {\dot{\mathbf{p}}} = {\mathbf{Kp}} $$

with the rate matrix

13 $$ {\mathbf{K}} = \left( {\begin{array}{*{20}c} { - k_{{{\text{ex}}}} } & {k_{D} } & {k_{A} } \\ {k_{{{\text{ex}}}} } & { - \left[ {k_{D} + k_{T} \left( r \right)} \right]} & 0 \\ 0 & {k_{T} \left( r \right)} & { - k_{A} } \\ \end{array} } \right) $$

Here, ${k}_{\mathrm{ex}}$ is the excitation rate of the donor, ${k}_{D}$ and ${k}_{A}$ are the decay rates of the excited states of donor and acceptor, respectively, and ${k}_{T}\left(r\right)={k}_{D}{\left({R}_{0}/r\right)}^{6}$ is the distance-dependent rate of energy transfer from donor to acceptor. The photon-rate of the donor as function of the distance is then given by $\widetilde{n}\left(r\right)={{\phi }_{D}k}_{D}{p}_{{D}^{*}A}^{ss}$ where the superscript $ss$ indicates the steady-state population that is obtained from $0=\mathbf{K}\mathbf{p}$ and ${\phi }_{D}$ is the quantum yield of the donor dye. As usual, the average donor photon rate is given by $\langle \widetilde{n}\rangle =\int \widetilde{n}\left(r\right)P\left(r\right)\mathrm{d}r$ such that the term $\delta \widetilde{n}\left(r\right)$ is directly accessible. Typical values of these parameters for the dye pair AlexaFluor 488 and AlexaFluor 594 are ${k}_{\mathrm{ex}}\approx 0.02 {\mathrm{n s}}^{-1}$, ${k}_{D}\sim {k}_{A}\approx 0.25 {\mathrm{n s}}^{-1}$, and ${\phi }_{D}=0.9$. Thus, Eqs. (12–13) together with the measured correlation time and a model for the distance distribution $P\left(r\right)$ can now be used to compute the intra-chain diffusion coefficient $D$. Once $D$ is known, also the reconfiguration time of the chain ${\tau }_{r}$ can be calculated using a similar approach, i.e., by replacing $\widetilde{n}\left(r\right)$ with $r$ in Eq. (12). Hence, reconfiguration times obtained using FRET-coupled nsFCS experiments depend on the model $P\left(r\right)$.

### Parameters required to determine the Rouse time

To estimate internal friction times from the measured reconfiguration times, we computed the Rouse time of the chain at various solvent conditions. To this end, the Kuhn segment size ${b}_{K}$ and the number of Kuhn segments ${N}_{K}$ were determined from the mean donor–acceptor distance ${R}_{\mathrm{DA}}$ at 6 M GdmCl (Table 3), i.e., conditions at which internal friction becomes negligible. These parameters are determined by the two relationships

14 $$ R_{{{\text{DA}}}}^{2} = b_{K}^{2} N_{K} = 2l_{p} bN_{{{\text{bonds}}}} \;{\text{and}}\;bN_{{{\text{bonds}}}} = b_{K} N_{K} . $$

**Table 3:** Parameters of the Rouse model obtained from mean donor–acceptor distance ${R}_{DA}$ and the reconfiguration time ${\tau }_{r}$ at 6 M GdmCl

	Myc	Max	Mad	ΔMyc
${N}_{K}$	18	18	18	18
${b}_{K}$	2.1	2.0	2.1	2.0
*a*	0.34	0.32	0.32	0.27

Units of the Kuhn length ${b}_{K}$ and the Stokes-radius $a$ of a bead in the Rouse chain are given in nm

Here, ${l}_{p}$ is the persistence length of the chain. Using Eq. (14), we obtain

15 $$ b_{K} = 2l_{p}  \;{\text{and}}\;N_{K} = \frac{{bN_{{{\text{bonds}}}} }}{{2l_{p} }}. $$

## Results

### The effect of denaturant on IDP dimensions

**Fig. 1:**
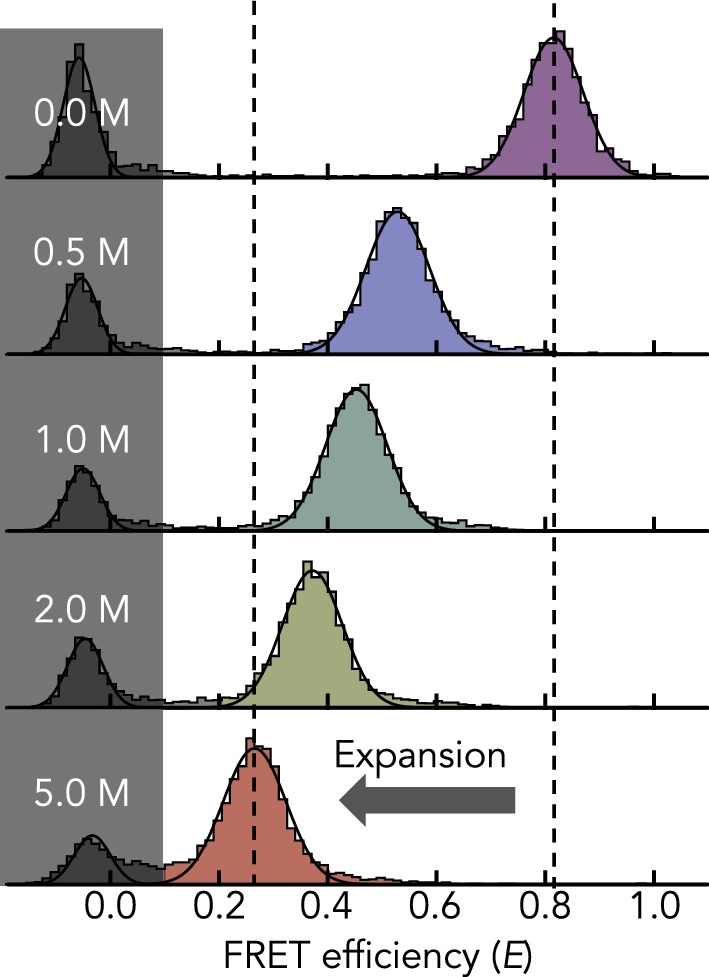
Examples of smFRET histograms of Max at increasing concentrations of GdmCl (indicated). Solid lines are fits with two Gaussian peaks. Dashed lines indicate the positions at 0 M and 5 M GdmCl. The decrease in FRET efficiency results from an expansion of the chain. The gray area indicates the molecules that lack an active acceptor dye

**Fig. 2:**
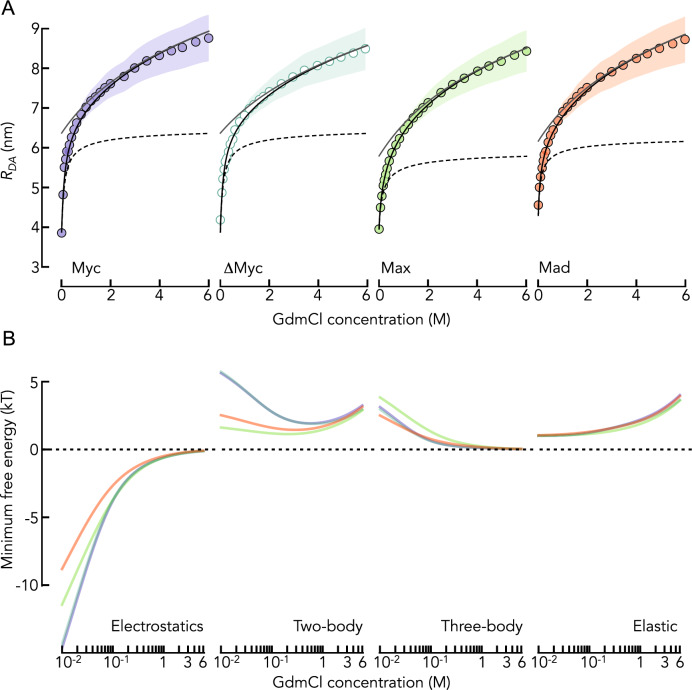
Energetic contributions to the expansion of Myc, Max, Mad, and ΔMyc. **A** Average donor–acceptor distance ${R}_{DA}$ of the IDPs (indicated) as function of the GdmCl concentration (circles). Shaded areas indicate the variation (standard deviation) due to the use of the polymer model (Eqs. 4–6). The black line is a fit with the polyampholyte theory (Eqs. 7–9). The black dashed line is the expansion due to a screening of electrostatic interactions by the ionic strength introduced by GdmCl. The solid gray line indicates the compaction due to a decrease in the two-body interaction parameter with decreasing GdmCl concentrations. **B** Contributions to the free energy (indicated) for the four IDPs (colors as in A: Myc (purple), Max (green), Mad (red), and ΔMyc (light green)). The free energy was computed at the measured average donor–acceptor distance ${R}_{DA}$

We used smFRET of the DNA-binding domains of the transcription factors Myc, Max, and Mad labeled with donor (AlexaFluor 488) and acceptor (AlexaFluor 594) fluorophores close to the chain termini (Table 1) to determine the average donor–acceptor distance (${R}_{\mathrm{DA}}$) at different concentrations of GdmCl. The FRET efficiency histograms of the three proteins show two peaks (Fig. 1). The peak close to zero FRET efficiency results from molecules that lack an active acceptor dye. The second peak at higher FRET efficiencies comes from donor–acceptor labeled molecules and reports on the mean transfer efficiency $\langle E\rangle$ from which we computed the mean donor–acceptor distance ${R}_{\mathrm{DA}}$ (Eqs. 3–6). As shown previously [4], ${R}_{DA}$ of all three proteins increases with increasing concentrations of GdmCl (Fig. 2A), thus reporting on the expansion of the chains due to the screening of electrostatic interactions at low GdmCl concentrations and increasing protein-solvent interactions at high GdmCl. We used an improved version of the polyampholyte theory of Higgs and Joanny [4, 6] to disentangle the energetic contributions to this expansion (Eqs. 7–10): electrostatic, two-body, and three-body interactions, and elastic entropy. Fits with the theory provide a good description of the data and allow us to estimate the contribution from the screening of electrostatic attractions to the overall chain expansion (Fig. 2B). Screening causes approximately 50% of the expansion of the chains within the accessible concentration range of GdmCl. We additionally investigated a variant of Myc that we term ΔMyc (Fig. 2A, Table 1), in which we replaced bulky hydrophobic residues (Val, Leu, Ile, Phe) by the amino acids Gly and Ser, thus lowering the hydrophobicity of Myc from 35 to 24% [42]. Neither the dimension ${R}_{DA}$ of ΔMyc under native conditions nor its GdmCl-induced expansion differed from the wild-type sequence (Fig. 2A), suggesting that hydrophobicity has a negligible role among the forces that dictate the dimension of Myc under native conditions. The contribution from electrostatic interactions to the overall dimension therefore seems to be the dominating feature in all variants, albeit to slightly lower extents in Max and Mad. In fact, a more detailed analysis of the individual contributions to the overall free energy of the chains shows differences between the proteins (Fig. 2B). For instance, electrostatic attractions are more pronounced in Myc and ΔMyc compared to Max and Mad. The reason for this difference originates from the charge composition of the chains. Positive and negative charges in the proteins are well mixed along the sequence such that only two sequence parameters are sufficient to describe electrostatic effects: the net charge per residues given by $f-g$ where $f$ and $g$ are the relative numbers of positively and negatively charged residues in the sequence, respectively, and the total fraction of charged residue given by $f+g$. The contribution of electrostatic repulsions to the free energy of the chain is proportional to ${\left(f-g\right)}^{2}$ and electrostatic attractions scale with ${\left(f+g\right)}^{2}$ (Eq. 7). Whereas ${\left(f-g\right)}^{2}\sim 0$ for all sequences, indicating negligible electrostatic repulsions, we find that ${\left(f+g\right)}^{2}$ is 0.18 for Myc and ΔMyc compared to 0.14 for Max and Mad. Hence, already based on sequence considerations, electrostatic attractions in Myc and ΔMyc are more than 25% higher than those in Max and Mad, which explains the increased contribution of electrostatic interactions to ${R}_{\mathrm{DA}}$ in Myc and ΔMyc (Fig. 2A). A more detailed comparison of all contributions to the free energy of the chains (Eq. 10) shows that the elastic free energy (chain entropy) is positive and increases with an expansion of the chain, as expected (Fig. 2B). Similarly, the contribution from repulsive three-body interactions diminishes with increasing expansion of the chains. Importantly, the free energy of two-body interactions is generally repulsive (> 0) but changes in a non-monotonic manner with increasing concentrations of GdmCl. At low concentrations, the free energy of two-body interactions first decreases with GdmCl, thus partially compensating the decrease in electrostatic attractions. At higher concentrations, the chain expansion is reflected in a linear increase in the two-body interaction parameter causes (Fig. 2B).

In summary, all chains are compact in the absence of GdmCl and expand with increasing GdmCl due to the screening of electrostatic attractions at low concentrations and by an increase in repulsive two-body interactions at high concentrations. The latter increase is presumably caused by an increase in protein-solvent interactions due to direct interactions of GdmCl with the chain [43].

### Dynamics in denaturant

**Fig. 3:**
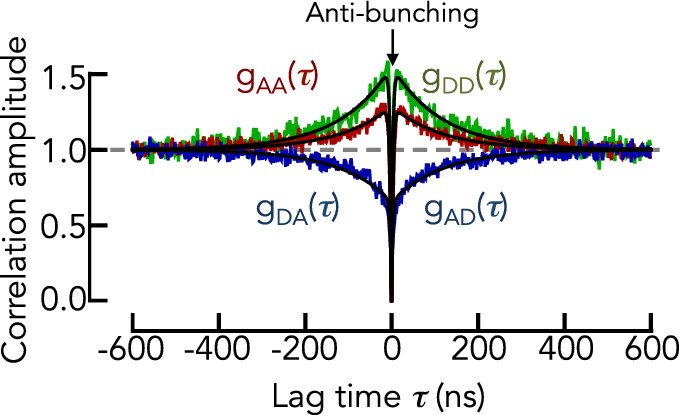
Normalized nsFCS traces of Myc at 0.2 M KCl. The donor (DD) and acceptor (AA) autocorrelation functions are shown in green and red, respectively. The cross-correlation functions (DA, AD) are shown in blue. The photon antibunching spike is indicated. Solid lines are the result of a global fit of the three correlation functions with Eq. (11)

**Fig. 4:**
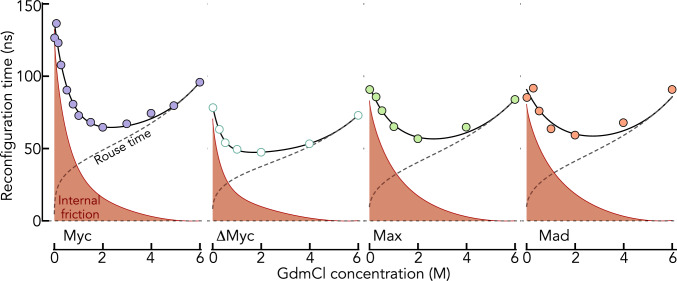
Reconfiguration times of the IDPs (indicated) as function of GdmCl. The solid line is an empirical interpolation fit. The gray dashed line indicates the Rouse time calculated by assuming that internal friction is negligible at 6 M GdmCl. The viscosity change due to the addition of GdmCl was taken into account in the computation of the Rouse time. According to the RIF model (Eq. 16), the difference between Rouse time and the experimental reconfiguration times is defined as internal friction (red area)

In a next step, we determined the reconfiguration dynamics of the IDPs at different concentrations of GdmCl using nsFCS. We find that the proteins show a dominant decay in the donor (${g}_{DD}$) and acceptor (${g}_{AA}$) autocorrelation functions and a rise in the cross-correlation functions (${g}_{AD/DA}$) (Fig. 3). The latter rise is due to the anti-correlated photon emission of donor and acceptor and is therefore indicative of donor–acceptor distance dynamics. The correlation time ${\tau }_{C}$ together with the determined donor–acceptor distance distribution then determines the reconfiguration time of the chain ${\tau }_{r}$ (Eqs. 12–13). Upon removal of GdmCl, ${\tau }_{r}$ first decreases and then increases again at low denaturant concentrations (Fig. 4). This non-monotonic behavior is the result of many effects that we empirically partition into only two contributions using the Rouse model with internal friction (RIF) [15, 16, 23, 32, 33, 44]. In RIF, the reconfiguration time is approximately^1^ given by two timescales, the Rouse time of the chain ${\tau }_{R}$ and an empirical internal friction timescale ${\tau }_{i}$

16 $$ \tau_{r} = \tau_{R} \frac{{\mathop \sum \nolimits_{z} A\left( {q,k,l} \right)/z^{4} }}{{\mathop \sum \nolimits_{z} A\left( {q,k,l} \right)/z^{2} }} + \tau_{i} \;{\text{with}}\;\tau_{R} = \frac{{N_{K} R_{{{\text{DA}}}}^{2} }}{{3\pi^{2} k_{B} T}}\xi_{S} $$

Here, $z=\left\{1, 2, 3,\dots ,{N}_{K}-1\right\}$ is the mode number, ${N}_{K}$ is the number of Kuhn segments in the chain (Eq. 15), $k$ and $l$ are the positions of the dyes in the sequence, ${\xi }_{S}=6\pi \eta a$ is the friction coefficient of a single Kuhn segment in the chain that depends on the solvent viscosity $\eta$ and the Stokes-radius $a$ of a Kuhn segment, ${k}_{B}$ is Boltzmann’s constant, and $q=z\pi /{N}_{K}$. RIF describes the chain as a coupled harmonic oscillator in the overdamped solvent regime. Chain monomers are coupled by harmonic springs (bonds), and internal friction is introduced as a local dissipative dampening process that independently acts on each bond. Naturally, the coupling of springs results in a spectrum of timescales whose weights depend on

17 $$ A\left( {q,k,l} \right) = \frac{2}{N}\left[ {\cos \left( {qk} \right) - \cos \left( {ql} \right)} \right]^{2} . $$

With decreasing dimension of the chain, i.e., decreasing concentration of GdmCl, the Rouse time will decrease as ${\tau }_{R}\propto {R}_{\mathrm{DA}}^{2}$, which, together with the decreasing viscosity of the solvent due to GdmCl removal, explains the decrease in reconfiguration times down to 2 M GdmCl (Fig. 4). To disentangle Rouse time and internal friction time, we assumed that internal friction is negligible at 6 M GdmCl; an assumption confirmed previously for several unfolded proteins [15, 16]. Using this reference point (Table 3), we computed the Rouse time as a function of the GdmCl concentration based on the change in ${R}_{\mathrm{DA}}$. Since ${R}_{\mathrm{DA}}$ decreases monotonically with decreasing concentrations of GdmCl, the increase in ${\tau }_{r}$ at very low GdmCl must be caused by internal friction (Fig. 4). As discussed in the introduction, the molecular origin of internal friction effects is currently unclear and a combination of dihedral angle rotations [23, 26], local and non-local interactions [27], as well as the crude approximation of harmonic bond potentials in the Rouse model [31] are in discussion [33]. It has previously been shown for different unfolded and intrinsically disordered proteins that ${\tau }_{i}$ increases with increasing compaction of the chain [15]. As the high compaction of Myc, Max, and Mad at 0 M GdmCl is mainly caused by strong attractive electrostatic interactions (Fig. 2B), the high ${\tau }_{i}$ for all proteins at native conditions (0 M GdmCl) should also be strongly impacted by these electrostatic interactions. In fact, the strength of these interactions is slightly lower in Max and Mad compared to Myc (Fig. 2B), which correlates with reduced internal friction times in these proteins, suggesting that electrostatics are indeed a dominant contribution to internal friction in these proteins. However, a comparison of Myc with ΔMyc shows that electrostatics is not the only contribution. Even though the strength of electrostatic interactions is very similar in Myc and ΔMyc, the internal friction time is nearly twofold lower in ΔMyc compared to Myc. Since ${R}_{\mathrm{DA}}$ of ΔMyc is similar to that of Myc at 0 M GdmCl, the difference in internal friction might either result from the reduced hydrophobicity of ΔMyc compared to Myc, or it might originate from the increased degrees of freedom due to the incorporation of Gly residues in the chain. Since our analysis of the energetic contributions to the chain dimensions did not reveal a substantial difference in the two-body interaction term between both sequences, a term that also includes hydrophobic effects, we conclude that electrostatic interactions and dihedral angle rotations are the main contributors to ${\tau }_{i}$ at low salt concentrations. To understand whether internal friction is impacted differently by intra-chain contacts caused by electrostatics or by the hydrophobic effect, we performed experiments in which we artificially increased the strength of the hydrophobic effect.

### Dissecting the contributions to internal friction

**Fig. 5:**
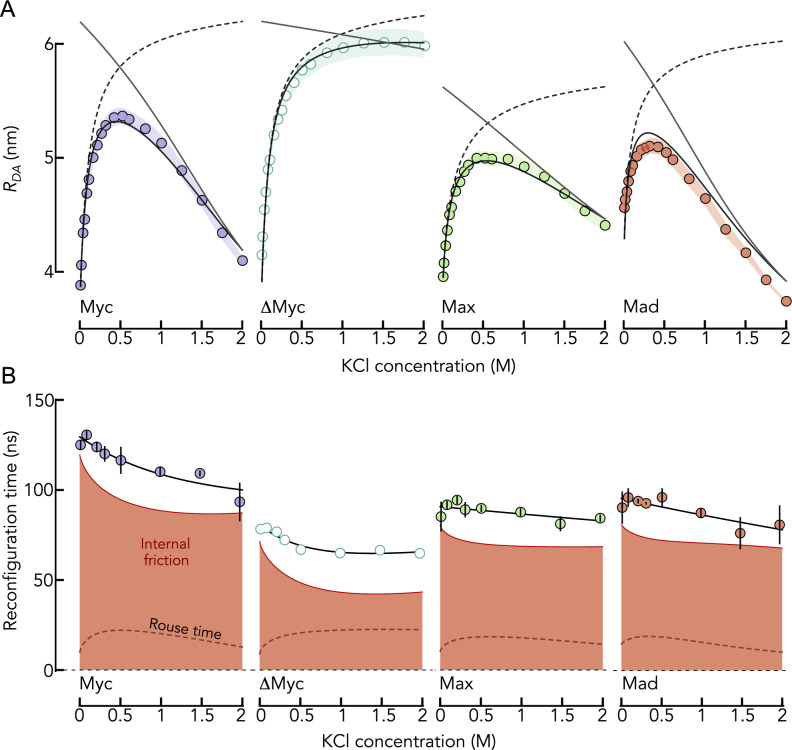
The effect of KCl on the dimension and dynamics of the IDPs. **A** Average donor–acceptor distance ${R}_{DA}$ of the IDPs (indicated) as function of the KCl concentration (circles). Shaded areas indicate the variation (standard deviation) due to the use of the polymer model (Eqs. 4–6). The black line is a fit with polyampholyte theory (Eqs. 7–9). The black dashed line is the expansion due to a screening of electrostatic interactions by the ionic strength introduced by KCl. The solid gray line indicates the effect of the two-body interaction parameter that becomes attractive at high KCl concentrations. **B** Reconfiguration times of the IDPs (indicated) as function of the KCl concentration. The solid line is an empirical interpolation fit. The gray dashed line indicates the Rouse time. The internal friction time is shown as red area. Error bars result from two independent experiments

**Fig. 6:**
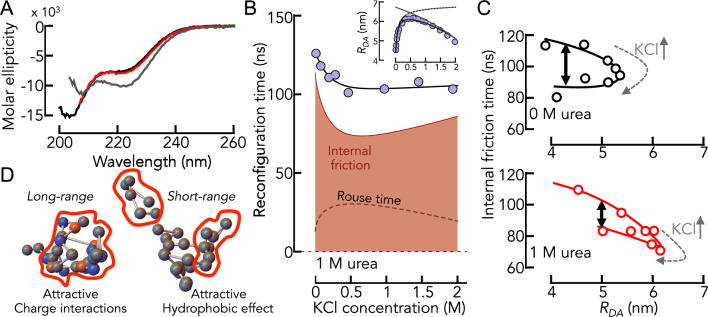
Electrostatics and hydrophobicity have different effects on internal friction. **A** CD-spectra of Myc in the absence of KCl (black), in the presence of 2 M KCl (gray) and in the presence of 2 M KCl and 1 M urea (red). **B** Reconfiguration times of Myc (circles) as function of KCl and in the presence of 1 M urea. Internal friction and Rouse time are indicated. Inset: Donor–acceptor distance as function of the KCl concentration at 1 M urea. Lines describe the contributions from screening (gray solid) and hydrophobic effect (gray dashed) and are described in Fig. 5A. **C** Correlation between internal friction time and average donor–acceptor distance ${R}_{DA}$ as function of KCl in the absence (top) and presence (bottom) of 1 M urea. A difference in internal friction time is observed at identical ${R}_{DA}$ but different KCl concentrations (black double arrow). **D** Schematic representation explaining the difference in C by long-range interactions at low KCl that lead to a slow-down in internal friction compared to short-ranged hydrophobic effects that are more prominent at high KCl concentrations

We demonstrated previously that an increase in the concentration of KCl causes Myc, Max, and Mad to expand due to a screening of electrostatic attractions, similar to the result found with GdmCl [4]. Yet, contrary to GdmCl, which is a chaotropic salt, KCl is a cosmotropic salt that increases the hydrophobic effect at high concentrations [4, 5]. Correspondingly, Myc, Max, and Mad compact at high (molar) concentrations of KCl (Fig. 5A). In result, the proteins form similarly compact conformational ensembles at low and at high KCl concentrations. This allows us to compare the dynamics of these proteins under conditions at which either electrostatic interactions (low KCl concentration) or hydrophobic effects (high KCl concentration) dominate. Indeed, our smFRET experiments show that ${R}_{\mathrm{DA}}$ first increases and then decreases with increasing concentrations of KCl. A fit with the polyampholyte theory shows that the initial increase results from a screening of attractive electrostatic interactions whereas the compaction at high KCl concentrations results from a decrease in the repulsive two-body interaction energy in the chain (Fig. 5A), which is a result of the increased hydrophobic effect [4]. Naturally, this compaction is absent for ΔMyc, in which we replaced bulky hydrophobic residues by Gly and Ser (Fig. 5A). When using nsFCS to determine the reconfiguration times of the chains, we found that contrary to the prominent and non-monotonic change in ${R}_{\mathrm{DA}}$, the reconfiguration time of the chains are only moderately impacted by the addition of KCl (Fig. 5B). The reconfiguration time of Myc drops from 125 ± 2 ns to 93 ± 10 ns across the whole range KCl concentrations. Similar changes, albeit even less pronounced were observed for ΔMyc, Max, and Mad (Fig. 5B). We then used the RIF model to extract the internal friction timescale for all proteins. We found that ${\tau }_{i}$ decreases with increasing KCl concentrations for all proteins. While this behavior was expected for the monotonically expanding ΔMyc variant in which the contributions from hydropbobic effect are negligible, the diminished ${\tau }_{i}$ for Myc, Max, and Mad at high KCl concentrations surprises. Since the internal friction time at high KCl concentrations is reduced compared to conditions at which electrostatics dominate (low KCl concentrations), one would naively conclude that electrostatic interactions have a stronger impact on internal friction than the hydrophobic effect at identical dimensions. This interpretation is corroborated by the stronger drop in internal friction for Myc and ΔMyc (Fig. 5B), i.e., sequences with stronger electrostatic attractions than Max and Mad (Fig. 2B). Yet, care has to be taken. High salt concentrations are known to stabilize secondary structures in proteins. We therefore measured the far-UV CD-spectrum of Myc at low and high KCl concentrations (Fig. 6A). Indeed, we found an increased helical signal at 2 M KCl, suggesting the formation of helical structures. To understand whether the dynamics of helix formation contributes to the dynamics of Myc, we repeated our experiments in the presence of the charge-neutral denaturant urea. Using CD-spectroscopy, we first confirmed that 1 M urea destabilizes helical secondary structures sufficiently at 2 M KCl, i.e., the same CD-spectrum of Myc is found in the absence of KCl and in 2 M KCl but in the presence of 1 M urea (Fig. 6A). We then repeated the smFRET and nsFCS experiments for Myc at 1 M urea (Fig. 6B). Importantly, albeit ${R}_{\mathrm{DA}}$ is slightly increased in the presence of urea due to increased protein-solvent interactions and a diminished hydrophobic effect; the expansion and compaction of Myc with increasing KCl concentration are preserved even in the presence of urea (Fig. 6C). Similarly, the decrease in reconfiguration time with increasing concentrations of KCl is preserved at 1 M urea (Fig. 6B). When we calculated the contribution from internal friction to the reconfiguration times, we found that ${\tau }_{i}$ first decreases and then slightly re-increases with increasing concentrations of KCl. Yet, a correlation map shows that even at the highest KCl concentration, ${\tau }_{i}$ does not reach the value found in the absence of KCl (Fig. 6C). Since we can rule out any impact from secondary structure formation at 1 M urea, the result suggests that both attractive electrostatic interactions and the attractive hydrophobic effect can cause internal friction in IDPs. Quantitatively however, electrostatic attractions seem to be more efficient in slowing down the dynamics of IDPs.

## Discussion

A substantial part of our understanding of IDPs is based on concepts from polymer physics. Length scaling exponents of IDPs for instance provide us with an absolute scale to measure the expansion and compaction of IDPs [39, 45–47]. Similarly, theories describing the coil-to-globule transition of homopolymers [48–51] have been successfully used to describe the response of unfolded and intrinsically disordered proteins to changes in external conditions such as temperature or solvent compositions [1, 4, 34, 52–56]. Mean-field theories of the same spirit, such as Flory–Huggins theory [57, 58], are in active use to describe the reversible formation of condensates of IDPs such as in liquid–liquid phase separation processes [59, 60]. The success of these theories is surprising given that IDPs are far from being homopolymers. Indeed, the number of cases in which homopolymer theories reach their limits in describing IDPs is increasing. The incorporation of the patterning of charges [9, 10] or hydrophobic residues [61] into existing theories [62], but also coarse-grained simulations [5] are now being used to describe the conformational ensemble of IDPs in which amino acids of different types are not well mixed along the sequence. In such cases, classical polyampholyte and polyelectrolyte (homopolymer) theories fail to describe the thermodynamic properties of IDPs. Yet, contrary to thermodynamics, no such deviations from homopolymer theories have so far been found for the dynamics of IDPs. Rouse-type models, or alternatively their improved Zimm-versions with hydrodynamic interactions, reproduce experimental data on IDPs and unfolded proteins well [15, 16, 33, 63]. We therefore asked whether the reconfiguration dynamics of IDPs is really independent of the actual types of interactions in these chains. Specifically, we asked whether attractive electrostatic interactions or the hydrophobic effect affect IDP dynamics differently. While electrostatic interactions are long-ranged, the hydrophobic effect is rather short-ranged (Fig. 6D) and depends on the solvation shells around bulky hydrophobic groups. Using nsFCS coupled with smFRET, we quantified the sub-microsecond dynamics of four intrinsically disordered sequences (Myc, Max, Mad, ΔMyc) at varying solvent conditions. We analyzed the obtained reconfiguration times of the chains using a Rouse model with an additional internal friction parameter. Our results show that a small but notable difference in the internal friction timescale can be observed between two conditions. At low salt concentrations, the chains are compact due to strong electrostatic attractions. At high salt concentrations however, the chains are compact due to a strong hydrophobic effect. Interestingly, the internal friction timescale is higher in the presence of strong electrostatic attractions (low salt), suggesting that reconfigurations of the chain are slower compared to conditions at which the hydrophobic effect dominates. This result might not surprise given the long-range nature of electrostatic interactions; the Debye-screening length at our lowest salt concentration (0.01 M) is ~ 3 nm, which is close to the end-to-end (donor–acceptor) distance of the sequences (~ 4 nm). Hence, even charges at the opposite termini of the chains will still interact. Notably, our results shed new light on the discussion of what precisely the origin of internal friction is [33, 64]. Both global electrostatic interactions but also local hydrophobic interactions clearly contribute. In addition, the investigation of a variant of Myc in which we replaced all bulky hydrophobic residues by serine and glycine allowed us to study the effect of dihedral angle rotations on the internal friction timescale. At the lowest salt concentration, ΔMyc has the same dimension as Myc due to similar electrostatic attractions and a negligible hydrophobic effect. The twofold difference in reconfiguration time and in internal friction time between the variants is clear evidence that dihedral angle rotations that are less restricted in ΔMyc contribute to internal friction.

In summary, our results show that different types of interactions affect the fast dynamics of IDPs differently. At identical dimension of the chains, hydrophobic interactions contribute less to internal friction than electrostatic interactions. Although these differences are small, they provide a first step toward disentangling the energetic contributions of the various interaction types in IDPs to their dynamics.

## Data Availability

The raw data in this work are available from the authors upon request.

## Footnotes

1 Only the correlation time of the end-to-end vector correlation function is analytically accessible in Rouse models, which is not exactly the same as the correlation time of the end-to-end distance measured in our experiments. For a comparison between both quantities see ref. 44.
